# Development of a Consensus-Based List of Potential Quality Indicators for Fever and Inflammation of Unknown Origin

**DOI:** 10.1093/ofid/ofad671

**Published:** 2024-01-11

**Authors:** William F Wright, Albrecht Betrains, Lauren Stelmash, Catharina M Mulders-Manders, Chantal P Rovers, Steven Vanderschueren, Paul G Auwaerter

**Affiliations:** Division of Infectious Diseases, Department of Medicine, School of Medicine, Johns Hopkins University, Baltimore, Maryland, USA; General Internal Medicine Department, University Hospitals Leuven, Leuven, Belgium; Division of Infectious Diseases, Department of Medicine, Johns Hopkins Bayview Medical Center, Baltimore, Maryland, USA; Division of Infectious Diseases, Department of Internal Medicine, Radboud University Medical Center, Nijmegen, the Netherlands; Division of Infectious Diseases, Department of Internal Medicine, Radboud University Medical Center, Nijmegen, the Netherlands; General Internal Medicine Department, University Hospitals Leuven, KU Leuven, Leuven, Belgium; Department of Microbiology, Immunology, and Transplantation, Laboratory of Clinical Infectious and Inflammatory Disorders, KU Leuven, Leuven, Belgium; Division of Infectious Diseases, Department of Medicine, School of Medicine, Johns Hopkins University, Baltimore, Maryland, USA; The Sherrilyn and Ken Fisher Center for Environmental Infectious Diseases, School of Medicine, Johns Hopkins University, Baltimore, Maryland, USA

**Keywords:** fever of unknown origin, inflammation of unknown origin, pyrexia of unknown origin, quality indicators

## Abstract

With a growing emphasis on value-based reimbursement, developing quality indicators for infectious diseases has gained attention. Quality indicators for fever of unknown origin and inflammation of unknown origin are lacking. An assembled group of international experts developed 12 quality measures for these conditions, which could be validated with additional study.

## Teaser

We sought to identify quality indicators for fever and inflammation of unknown origin, which are currently lacking. Sixteen experts offered 40 suggestions organized into 9 general categories. A list of 12 agreed-on potential quality indicators was established that could inform the care of these patients with further study.

Balancing health system costs and quality against population needs has become a priority for health care leaders and clinicians in the United States and globally [1]. The Infectious Diseases Society of America’s Clinical Affairs Committee has sought to quantify the value of infectious diseases (ID) trained physicians to the health care system by promoting quality improvement and measures in the areas of patient care, infection prevention, and antimicrobial stewardship [2–4]. Evidence in the literature has demonstrated that ID physician interventions are associated with improved diagnostic accuracy and outcomes, reduced length of hospital admissions and costs, fewer therapy-related complications, and reduced *Staphylococcus aureus* bacteremia-associated mortality rates [2–4]. With the shift to value-based reimbursement, well-defined quality indicators are needed to translate this evidence of quality and performance measures for patient care [2–4]. ID physicians often serve as consultants for patients with classic fever or inflammatory conditions of unknown origin (FUO and IUO, respectively). They are well positioned to develop a robust set of metrics that lead to improved patient care and associated costs of evaluation [5].

Classic FUO was prospectively defined in 1961 by Petersdorf and Beeson in a hospital-based study, in which the definition included a temperature ≥100.9 °F (≥38.3 °C) on several occasions with an illness of >3 weeks and an uncertain diagnosis after 1 week [5, 6]. Subsequent investigators modified these criteria to reflect practice changes in medicine, such as the move toward outpatient evaluations and qualitative defining criteria [5, 6]. FUO and IUO are separate but closely related conditions with similar diagnostic criteria and etiologies [7]. In 2009, Vanderschueren et al [7] defined IUO as a condition lasting >3 weeks without high-grade fevers (ie, <38.3 °C), C-reactive protein levels >30 mg/L, and/or erythrocyte sedimentation rates greater than age divided by 2 in males or (age + 10) divided by 2 in females on >3 occasions, with no diagnosis despite appropriate investigations after at least 3 outpatient visits or at least 3 days in the hospital [7].

Quality measures for some infections were advanced by Eby et al [3] in 2019. Twenty-seven metrics were described, including standards for evaluating these candidate measures. According to this group, developing a composite of ID-specific quality process measures based on patient-level outcome data within many qualified clinical data registries would improve performance, promote excellence, and allow comparability across health care systems. Unfortunately, ID specialty–specific quality improvements are challenging, given the heterogeneity of patient populations and infectious conditions. Despite these difficulties, the Infectious Diseases Society of America has provided resources designed to help ID physicians formulate quality improvements and reduce barriers toward advancing a value-driven health care system [4, 8].

FUO and IUO are ID specialty–specific conditions that lack uniform quality indicators. These conditions also lack diagnostic standards or rigorous prospective clinical trial evidence to make well-informed recommendations for developing quality indicators. Herein, we sought to start filling these gaps by engaging an international expert panel through the Delphi technique that could be beneficial for improving health outcomes.

## METHODS

This research is part of a modified 5-round Delphi technique that seeks to develop a consensus on current research and practice trends for classic FUO and IUO [9]. While the first 3 rounds were devoted to reaching a consensus on the diagnostic criteria and clinical evaluation of patients with these conditions, developing a list of potential quality indicators occurred during the fourth-round questionnaire.

The Delphi technique is structured to create consensus on topics when gaps exist in the available evidence [10, 11]. The selection of experts and design of the Delphi round for this study were applied in compliance with the CREDES standards (Conducting and Reporting of Delphi Studies) [11].

### Selection of Panelists

We surveyed a 21-member panel (Supplementary Table 1) representing researchers and clinician experts who routinely participate in the care and research of patients with FUO and IUO as evidenced by peer-reviewed published articles within the past 20 years.

### Questionnaire

This questionnaire was administered anonymously via RedCap. The link and instructions were provided to the panel of experts by secure electronic mail. Expert panelists were surveyed for their clinical opinions based on their previous experiences and interpretation of the available literature for the following question: “If you could choose a quality clinical metric for FUO and IUO outcomes, what would you choose and why?” The panel members were unaware of the identity and opinions of other panelists to not be influenced by others during the process.

### Institutional Review Board Review

This survey questionnaire was approved by the Johns Hopkins University School of Medicine Institutional Review Board and Office of Research Assistance (IRB00326408).

### Patient Consent Statement

This study does not include factors necessitating patient consent, but each survey participant agreed to written informed consent.

## RESULTS

### Characteristics of Panel Members

Twenty-one potential panelists were contacted in round 4, and 16 agreed to participate (Supplementary Table 1). Respondents included 8 ID physicians, 7 internal medicine physicians, and 1 pathologist. Twelve (75%) respondents were men, and 4 (25%) were women. All respondents practiced in academic medical centers.

### Establishing Quality Metrics for FUO and IUO

Respondents provided 40 statements within 9 general categories to describe potential quality indicators for these conditions (Table 1). We then participated in 2 in-person video meetings (eg, Delphi rounds 4a and 4b) to discuss the group consensus and develop a final list of proposed quality indicators that reflected a combined consensus of the respondents and researchers of this study (Table 2).

**Table 1.** Proposed Quality Indicators From Respondents

Quality Indicator Themes	No. of Respondents Proving the Recommendation	Rating Based on the Total No. of Respondents (N = 16), %^a^
1. Time between symptom onset and diagnosis or effective treatment.	14	87.50
2. Time to spontaneous resolution of fever or inflammation for patients remaining undiagnosed.	10	62.50
3. Efficacy of evaluation methods (eg, diagnostic tests) to achieve a diagnosis	4	25.00
4. All-cause mortality (eg, survival at 30 d and 1 y) from time of meeting FUO or IUO criteria.	4	25.00
5. No. of visits before diagnosis (eg, hospital days, outpatient, or emergency ward).	3	18.75
6. Quality-of-life outcomes from meeting FUO or IUO criteria to a diagnosis.	2	12.50
7. No. of subspecialist consults required since referral for FUO or IUO to a diagnosis.	1	6.25
8. Objective verification of fever or inflammation markers for patients to meet FUO or IUO criteria.	1	6.25
9. FUO and IUO should merge as 1 condition.	1	6.25

Abbreviations: FUO, fever of unknown origin; IUO, inflammation of unknown origin.

^a^Number of respondents providing the recommendation divided by the total number of respondents (N = 16).

**Table 2.** Panel of Proposed Quality Indicators for Assessment, Management, and Follow-up in Fever and Inflammation of Unknown Origin Expert Centers

Time: Quality Indicator Theme	Definition	Evaluation Method	Metric Type	Quality Domain
Preevaluation				
Referral triage	No. of triaged patients and the length of time between referral and initial consultation at a center with experienced physicians	No. of FUO and IUO referrals triaged per month or year and time from referral to initial consultation	Structure	Access
Center volume	No. of evaluations performed	No. of FUO and IUO evaluations performed per month or year	Structure	Access
Physician expert^a^	No. of FUO and IUO physician experts	No. of FUO and IUO physician experts per center	Structure	Access
Physician diversity	No. of specialty and subspecialty physicians available to assist with evaluations	No. of subspecialist physicians available to assist with evaluations per center	Structure	Access
Evaluation				
Hospital length of stay	No. of days in the hospital among patients requiring hospitalization from day of admission to day of discharge after evaluation, with each stay past midnight counting as a hospital day	Median length of hospital stays among patients requiring hospitalization for patients with and without a final diagnosis	Outcome	Effectiveness
No. of inpatient contacts	No. of inpatient evaluations with and without a final diagnosis	Median inpatient visits for patients with and without a final diagnosis	Outcome	Effectiveness
No. of outpatient contacts	No. of outpatient evaluations with and without a final diagnosis	Median outpatient visits for patients with and without a final diagnosis	Outcome	Effectiveness
Laboratory resource utilization	No. of laboratory tests used to assist with evaluations	No. of laboratory tests used per patient by diagnosis	Process	Safety and effectiveness
Radiologic resource utilization	No. of radiology tests used to assist with evaluations	No. of radiologic tests used per patient by diagnosis	Process	Safety and effectiveness
Invasive procedure or biopsy utilization	No. of invasive procedures or biopsies used to assist with evaluations	No. of invasive procedures or biopsies used per patient by diagnosis	Process	Safety and effectiveness
Total time to diagnosis	Time from initial consultation to the time of reaching a definitive diagnosis.	Time required to reach a definitive or probable diagnosis per patient	Process	Safety and effectiveness
Postevaluation care and outcome				
1-y mortality	Percentage of patients who died within the first year post-FUO/IUO evaluation with and without a final diagnosis	Kaplan-Meier survival statistical analysis	Outcome	Effectiveness

Abbreviations: FUO, fever of unknown origin; IUO, inflammation of unknown origin.

^a^The definition of a physician expert is based on the American Medical Association’s *Principles of Medical Ethics* opinion 9.7.1, in which a physician expert is defined as having appropriate training and recent substantive experience and knowledge that reflect current scientific thought and standards of care that have gained acceptance among peers in the relevant field (https://code-medical-ethics.ama-assn.org/sites/default/files/2022-08/9.7.1.pdf).

## DISCUSSION

In this study, we aimed to develop quality indicators for FUO and IUO by engaging an international expert panel through the Delphi technique. We identified 40 recommendations, 8 quality indicator categories, a final list of 12 potential quality indicators, and 1 recommendation to merge FUO and IUO into 1 condition. Although merging is a worthy consideration, it falls beyond this study's intent and would require further study to understand whether consolidating or keeping these conditions independent would facilitate patient care.

### Implications for Practice

The utility of a quality indicator in routine practice depends on its role in guiding clinical decisions that could affect patient outcomes. Outcome measures have been the preferred approach for developing quality infectious disease indicators because process measures are not always a perfectly mapped surrogate for the desired result, particularly among conditions with significant heterogeneity such as FUO and IUO [2, 12]. For example, educating clinicians on utilizing protocolized (algorithmic) FUO or IUO diagnostic evaluations does not necessarily result in the reduction of patients remaining undiagnosed, nor is physician education the only way to improve achieving a diagnosis [13]. Outcome measures can also be supplemented with statistical methods, such as stratification or risk adjustment, that control for factors that influence the relationship between a predictor and outcome that is often out of the clinician's control [14, 15]. An example of this would be the underutilization of sophisticated diagnostic techniques, such as ^18^FDG-positron emission tomography/computed tomography and molecular diagnostics, in some geographic locations with fewer medical resources [16].

The outcome measures considered most important by respondents in this study to promote quality improvement were the time between symptom onset and diagnosis or effective treatment and the time to spontaneous resolution of fever or inflammation for undiagnosed patients (Table 1). Instead of the symptom onset date, which may be nonspecific and overlap with other underlying conditions that may or may not be related to fever or inflammation, we suggest refinement of this metric to a more objective time point, such as (1) the time of initial referral to a center with expert FUO/IUO clinicians or (2) the time from initial consultation appointment to a definitive diagnosis. The center's effectiveness would be reflected in the first, while the second would be related to the expert clinician's efficiency.

For patients receiving therapeutic agents for presumptive clinical diagnoses (eg, anakinra for an undifferentiated inflammation or empiric standard 4-drug therapy for tuberculosis), we propose that they should have appropriate metrics on the time points from initial consultation and follow-up for the resolution that is appropriate for the underlying presumptive condition. In this fashion, whether treatment is successful, supporting a clinical diagnosis, or sufficiently improves patients’ health without complication would be factored into determining if an approach is successful [17–20]. Finally, while the spontaneous resolution of fever or inflammation marks a vital milestone among patients with undiagnosed FUO/IUO, since this phenomenon is out of the clinician's control, it should not be advanced as a proposed quality metric but worthy of capturing for research purposes.

Given the intent of this study to promote the quality of patient-centered care, it is reassuring that experts recommended quality indicators for evaluation methods, empirical treatments, and survival. Whether to pursue empiric therapy (eg, antimicrobial agents or corticosteroids) for presumptive diagnoses should be only when a patient's health is at risk for severe deterioration or diagnostic evaluation has been exhausted.

An obvious question with FUO or IUO is whether an objective fever measurement is necessary. In the regular clinical care of a patient exhibiting high inflammatory markers or anemia, we contend that confirmation of fevers is not required and that patient report of fevers being present or absent is satisfactory. Waiting for a medical professional to record fever in these situations is unlikely to affect the patient's course of care or outcome. However, in the clinical setting of normal inflammatory markers or absence of anemia, fevers should be verified before subjecting patients to extensive evaluations or a research protocol. The proposed measures reflect the pragmatic nature of data, which should be readily available in the routine management of these patients.

Recommendations on the quality of clinicians and centers conducting these evaluations were absent. Rather, quality indicators reflecting patient complexity and decision making would be best compiled to reflect a given center, since a clinician may have a focused set of patients to evaluate or be subject to other factors (eg, a senior clinician seeing more patients who are seeking second and third opinions; Table 2) [3]. In other words, adjustment of these metrics could be based on a center—for example, one primarily evaluating cases of lower complexity whose patients appear to perform better than those in centers evaluating highly complex cases [3]. Additional items include resource utilization (eg, laboratory and radiology tests, invasive procedures), the number of inpatient and outpatient encounters required to arrive at a presumptive or final diagnosis, and hospital length of stay (if hospitalization is required). These outcomes must, however, first and foremost be validated as well as account for issues of case mix biases and complexity (ie, account for differences among diagnoses, severity of disease, and institutions) and avoid institutional stigma (ie, discriminate so-called good vs bad performers) [3, 12].

Finally, quality of life is a subjective compilation of assessments made by patients to describe their experiences with health and illness, which can vary among individuals and institutions and does not replace evaluations of specific illness outcomes [21–23]. Quality of life is also challenging to apply in clinical practice without using standardized instruments such as the SF-36 or the WHOQOL-BREF from the World Health Organization [21–23]. Furthermore, these standardized instruments are unable to produce a reliable single index of health-related quality of life. They likely would produce skewed results due to conditions with fluctuating health states, the timing of assessments during disease, and unreliability with standard recall periods (eg, “past 4 weeks” for SF-36) that could lead to systematic biases and misleading results [22, 23]. Therefore, we did not incorporate the quality-of-life recommendation into the final list of proposed indicators.

In general, the proposed quality indicators developed from this study may serve as a start to improving the quality of care for patients with FUO and IUO. After these quality indicators have been studied and validated, they might be helpful to health care organizations and administrators to gauge ID physician–led center performance in the value-oriented domains of effectiveness and safety. FUO and IUO physician experts may seek to improve quality by monitoring and optimizing operations within their centers using these indicators. Public knowledge of these quality indicators would help patients choose where to seek care [12].

### Implications for Research

Further development of clinical quality concepts identified in this study should be studied for their scientific merits by using the Centers for Medicare & Medicaid Services’ Quality Data Model, regarded as the gold standard method [14, 15]. The process of creating clinical quality indicators would be expected to be complex and time-consuming and involve numerous stakeholders. Proposed quality indicators from this study would first need to be assessed for feasibility to ensure data availability, accuracy, and workflow in routine clinical practice with minimal unintended consequences, such as increasing clinician and patient burdens, implementation costs, or clinical workflow changes [14, 15].

Once any of the quality indicators from this study are deemed feasible, proper evaluation should ensure scientific validity and reliability [14, 15]. A quality indicator, at its most basic level, is a ratio where the numerator is the number of clinical actions of interest performed and the denominator is the number of qualifying patients. Therefore, testing based on signal (numerator action) to noise (random variability and error), interrater reliability with a reported kappa statistic, and test-retest reliability are all appropriate measurement techniques [14, 15]. Finally, once a scientifically accepted quality indicator has been validated and implemented in clinical practice, it would be necessary to regularly reevaluate it when new evidence changes best practices for FUO and IUO clinical care.

FUO and IUO are conditions without defined optimal evaluation and management strategies [5, 13]. To date, no high-quality randomized controlled trials have been performed [5]. Therefore, a trial design of quality indicators comparing management strategies by DOOR end points (desirability of outcome ranking) could provide pragmatic and patient-centered information that clinicians need in order to make informed decisions about patient care [24, 25]. The development of novel DOOR end points could be developed through a survey of clinician-scientists and clinical trialists using case vignettes [24, 25].

### Limitations

Our study has limitations. First and most important, the quality indicators proposed in this study have not been validated and will need further investigation. Second, we developed a list of quality indicators with subjectivity, given the less-than-robust available data. However, the Delphi method is a well-acknowledged strategy to approach a situation inherent in this study, where there is insufficient evidence to make recommendations [10, 11]. Finally, the results that we present are subject to variations within geographic locations and societies regarding the use of health care resources [13].

## CONCLUSIONS

Measuring quality and outcomes for patients with FUO and IUO offers a start to future research and care improvements, potentially driving future efficiency. Given the infrequency of these conditions, (1) evaluation by experts with knowledge and experience within a dedicated referral center and (2) timely access to specialized diagnostic methods should help improve outcomes if these metrics are adopted. It will be worthwhile to study if this lowers per capita costs. The proposed metrics must be further vetted through carefully designed future investigations.

## Supplementary Data

Supplementary materials are available at *Open Forum Infectious Diseases* online. Consisting of data provided by the authors to benefit the reader, the posted materials are not copyedited and are the sole responsibility of the authors, so questions or comments should be addressed to the corresponding author.

## Supplementary Material

ofad671_Supplementary_Data

## Notes

***Acknowledgments.*** We wish to thank Joshua C. Eby, MD (Division of Infectious Diseases and International Health, University of Virginia, Charlottesville, Virginia, USA), and Cornelius J. Clancy, MD (Department of Medicine, University of Pittsburgh, Pittsburgh, Pennsylvania, USA), for their critical review and valuable suggestions with the preparation of this manuscript.

***Author contributions*.** All authors meet guidelines set forth by the International Committee of Medical Journal Editors and take responsibility for the integrity of the data and the accuracy of the data analysis. Concept and design: W. F. W., P. G. A. Acquisition, analysis, or interpretation of primary data: W. F. W., L. S. Drafting of the manuscript: W. F. W. Critical revision of the manuscript for important intellectual content: W. F. W., A. B., L. S., C. M. M.-M., C. P. R., S. V., P. G. A. Statistical analysis: W. F. W., L. S. Administrative, technical, or material support: W. F. W., L. S., P. G. A. Senior-level faculty: C. M. M.-M., C. P. R., S. V., P. G. A. Fellow, Infectious Diseases Society of America: P. G. A.

***Originality of content*.** We verify that all information and materials in the article are original.

***Financial support*.** This work was supported by the Johns Hopkins University Sherrilyn and Ken Fisher Center for Environmental Infectious Diseases.

## References

[1] Berwick DM , Nolan TW, Whittington J. The triple aim: care, health, and cost. Health Aff (Millwood) 2008; 27:759–69. 18474969 10.1377/hlthaff.27.3.759

[2] McQuillen DP , MacIntyre AT. The value that infectious diseases physicians bring to the healthcare system. J Infect Dis 2017; 216(suppl 5):S588–93. 28938046 10.1093/infdis/jix326 PMC7107418

[3] Eby JC , Lane MA, Horberg M, et al How do you measure up: quality measurement for improving patient care and establishing the value of infectious diseases specialists. Clin Infect Dis 2019; 68:1946–51. 30256911 10.1093/cid/ciy814

[4] Sheridan KR , Lane MA, Kim TJ, Eby JC. Infectious disease providers’ knowledge of and engagement in quality improvement. Open Forum Infect Dis 2021; 8:ofab515. 35559129 10.1093/ofid/ofab515 PMC9088501

[5] Wright WF , Mulders-Manders CM, Auwaerter PG, Bleeker-Rovers CP. Fever of unknown origin (FUO)—a call for new research standards and updated clinical management. Am J Med 2022; 135:173–8. 34437835 10.1016/j.amjmed.2021.07.038

[6] Petersdorf RG , Beeson PB. Fever of unexplained origin: report on 100 cases. Medicine (Baltimore) 1961; 40:1–30. 13734791 10.1097/00005792-196102000-00001

[7] Vanderschueren S , Del Biondo E, Ruttens D, Van Boxelaer I, Wauters E, Knockaert DD. Inflammation of unknown origin versus fever of unknown origin: two of a kind. Eur J Intern Med 2009; 20:415–8. 19524186 10.1016/j.ejim.2009.01.002

[8] Infectious Diseases Society of America . ID-specific MIPS quality resources. Available at: http://www.idsociety.org/clinical-practice/quality-improvement/id-specific-mips-quality-resources/. Accessed March 2023.

[9] Wright WF , Wang J, Auwaerter PG. Investigator-determined categories for fever of unknown origin (FUO) compared with *International Classification of Diseases–10* classification of illness: a systematic review and meta-analysis with a proposal for revised FUO classification. Open Forum Infect Dis 2023; 10:ofad104. 36949875 10.1093/ofid/ofad104 PMC10026547

[10] Hasson F , Keeney S, McKenna H. Research guidelines for the Delphi survey technique. J Adv Nurs 2000; 32:1008–15. 11095242

[11] Jünger S , Payne SA, Brine J, Radbruch L, Brearley SG. Guidance on Conducting and Reporting Delphi Studies (CREDES) in palliative care: recommendations based on a methodological systematic review. Palliat Med 2017; 31:684–706. 28190381 10.1177/0269216317690685

[12] Lilford RJ , Brown CA, Nicholl J. Use of process measures to monitor the quality of clinical practice. BMJ 2007; 335:648–50. 17901516 10.1136/bmj.39317.641296.AD PMC1995522

[13] Wright WF , Betz JF, Auwaerter PG. Prospective studies comparing structured vs nonstructured diagnostic protocol evaluations among patients with fever of unknown origin: a systematic review and meta-analysis. JAMA Netw Open 2022; 5:e2215000. 35653154 10.1001/jamanetworkopen.2022.15000 PMC9164007

[14] Centers for Medicare & Medicaid Services, Office of the National Coordinator for Health Information Technology. Quality data model, version 5.6. 1 January 2021. Available at: https://ecqi.healthit.gov/qdm. Accessed June 2023.

[15] Centers for Medicare & Medicaid Services, Measures Management Systems. Risk adjustment in quality measurement. 1 September 2022. Available at: https://mmshub.cms.gov/tools-and-resources/mms-supplemental-materials. Accessed June 2023.

[16] Chen JC , Wang Q, Li Y, et al Current situation and cost-effectiveness of 18F-FDG PET/CT for the diagnosis of fever of unknown origin and inflammation of unknown origin: a single-center, large-sample study from China. Eur J Radiol 2022; 148:110184. 35121332 10.1016/j.ejrad.2022.110184

[17] Bleeker-Rovers CP , Vos FJ, de Kleijn EMHA, et al A prospective multicenter study on fever of unknown origin: the yield of a structured diagnostic protocol. Medicine (Baltimore) 2007; 86:26–38. 17220753 10.1097/MD.0b013e31802fe858

[18] Mulders-Manders CM , Pietersz G, Simon A, Bleeker-Rovers CP. Referral of patients with fever of unknown origin to an expertise center has high diagnostic and therapeutic value. QJM 2017; 110:793–801. 29036369 10.1093/qjmed/hcx158

[19] Betrains A , Wright WF, Moreel L, Staels F, Blockmans D, Vanderschueren S. Etiological spectrum and outcome of fever and inflammation of unknown origin: does symptom duration matter? Eur J Intern Med 2022; 106:103–10. 36261311 10.1016/j.ejim.2022.10.002

[20] Betrains A , Moreel L, Wright WF, Blockmans D, Vanderschueren S. Association between diagnostic outcomes and symptom pattern in fever and inflammation of unknown origin. Eur J Intern Med 2023; 115:157–9. 37296004 10.1016/j.ejim.2023.05.030

[21] Higginson IJ , Carr AJ. Measuring quality of life: using quality of life measures in the clinical setting. BMJ 2001; 322:1297–300. 11375237 10.1136/bmj.322.7297.1297 PMC1120388

[22] Lins L , Carvalho FM. SF-36 total score as a single measure of health-related quality of life: scoping review. SAGE Open Med 2016; 4:2050312116671725. 10.1177/2050312116671725 PMC5052926 27757230

[23] Sanghera S , Walther A, Peters TJ, Coast J. Challenges in using recommended quality of life measures to assess fluctuating health: a think-aloud study to understand how recall and timing of assessment influence patient responses. Patient 2022; 15:445–57. 34854064 10.1007/s40271-021-00555-7 PMC9197908

[24] Evans SR , Rubin D, Follmann D, et al Desirability of outcome ranking (DOOR) and response adjusted for duration of antibiotic risk (RADAR). Clin Infect Dis 2015; 61:800–6. 26113652 10.1093/cid/civ495 PMC4542892

[25] Doernberg SB , Tran TTT, Tong SYC, et al; Antibacterial Resistance Leadership Group. Good studies evaluate the disease while great studies evaluate the patient: development and application of a desirability of outcome ranking endpoint for *Staphylococcus aureus* bloodstream infection. Clin Infect Dis 2019; 68:1691–8. 30321315 10.1093/cid/ciy766 PMC6495020

