# Supplementary material for: Development of a Consensus-Based List of Potential Quality Indicators for Fever and Inflammation of Unknown Origin
Source: Open Forum Infect Dis. 2024 Jan 11;11(2):ofad671. doi: 10.1093/ofid/ofad671 (PMC10853001; doi:10.1093/ofid/ofad671)
Supplement: ofad671_Supplementary_Data [file ofad671_supplementary_data.docx]

**Supplemental Data and Tables**

| **Supplemental Table 1. Characteristics of the Delphi Survey Respondents for this Questionnaire** | | |
| --- | --- | --- |
| **Characteristic** | **No. (%) of Respondents*** | |
|  | **Polled**  **(n=21)** | **Responded**  **(n=16)** |
| **Gender** |  | |
| Males | 15 (71.4) | 12 (75.0) |
| Females | 6 (28.6) | 4 (25.0) |
| **Age, year** |  | |
| 25-34 | 1 (4.8) | 1 (6.3) |
| 35-44 | 3 (14.3) | 2 (12.4) |
| 45-54 | 9 (42.9) | 8 (50.0) |
| 55-65 | 6 (28.6) | 4 (25.0) |
| Over 65 | 1 (4.8) | 1 (6.3) |
| **Specialty** |  | |
| Infectious diseases | 10 (47.6) | 8 (50.0) |
| Internal medicine | 9 (42.9) | 7 (43.7) |
| Pathology | 1 (4.8) | 1 (6.3) |
| Radiology | 1 (4.8) | 0 (0) |
| **Setting** |  | |
| Academic hospital | 17 (80.9) | 16 (100.0) |
| Community hospital | 4 (19.1) | 0 (0) |
| *The data on years of physician experience among the 16 respondents, as analyzed by the Shapiro-Wilk test, was normally distributed with **W** = 1.02 (reference p=0.887). Therefore, the mean years of experience was 22.0 (standard deviation 9.4).  All data and demographics were analyzed with Stata version 17 using the metaprop command for statistical analysis (StataCorp 2022, Stata Statistical Software: Release 17; StataCorp LLC, College Station, TX).  Reference for the Shapiro-Wilk test: Mishra P, Pandey CM, Singh U, Gupta A, Sahu C, Keshri A. Descriptive statistics and normality tests for statistical data. Ann Card Anaesth. 2019 Jan-Mar;22(1):67-72. doi: 10.4103/aca.ACA_157_18. | | |
